# Case Report: A rare treatable metabolic syndrome (Brown-Vialetto-Van Laere syndrome) masquerading as chronic inflammatory demyelinating polyneuropathy from Saudi Arabia

**DOI:** 10.3389/fped.2024.1377515

**Published:** 2024-04-30

**Authors:** Amal Y. Kentab, Yara Alsalloum, Mai Labani, Abrar Hudairi, Muddathir H. Hamad, Dima Z. Jamjoom, Ali H. Alwadei, Reem M. Alhammad, Fahad A. Bashiri

**Affiliations:** ^1^ Division of Pediatric Neurology, Department of Pediatrics, College of Medicine, King Saud University, Riyadh, Saudi Arabia; ^2^ Department of Pediatrics, King Saud University Medical City, Riyadh, Saudi Arabia; ^3^ Pediatric Intensive Care Unit, Department of Pediatrics, King Khalid University Hospital, King Saud University Medical City, Riyadh, Saudi Arabia; ^4^ Department of Radiology and Medical Imaging, College of Medicine, King Saud University, Riyadh, Saudi Arabia; ^5^ Pediatric Neurology Department, National Neuroscience Institute, King Fahd Medical City, Riyadh, Saudi Arabia; ^6^ Department of Internal Medicine, College of Medicine, King Saud University, Riyadh, Saudi Arabia

**Keywords:** pontobulbar palsy, riboflavin transporter deficiency, Brown-Vialetto-Van Laere syndrome, MRI, chronic inflammatory demyelinating polyneuropathy

## Abstract

**Background:**

Brown-Vialetto-Van Laere (BVVL) syndrome is an extremely rare autosomal recessive progressive motoneuron disease that is caused by a defect in the riboflavin transporter genes SLC52A2 and SLC52A3. BVVL syndrome has a variable age of presentation, and it is characterized by progressive auditory neuropathy, bulbar palsy, stridor, muscle weakness, and respiratory compromise secondary to diaphragmatic and vocal cord paralysis. BVVL syndrome has a poor prognosis in the absence of treatment, including morbidity with quadriparesis and sensorineural hearing loss, with mortality in the younger age group. Early administration of riboflavin is associated with prolonged survival, low morbidity, and reversal of some clinical manifestations.

**Case presentation:**

We describe an 18-month-old male infant with progressive pontobulbar palsy, loss of developmental milestones, and a clinical picture suggestive of chronic inflammatory demyelinating neuropathy. A nerve conduction study revealed axonal neuropathy, while molecular analysis revealed a homozygous mutation in one of the riboflavin transporter genes, SLC52A3, confirming BVVL syndrome. The patient needed long-term respiratory support and a gastrostomy tube to support feeding. With high-dose riboflavin supplementation, he experienced moderate recovery of motor function.

**Conclusion:**

This report highlights the importance of considering BVVL syndrome in any patient who presents with the clinical phenotype of pontobulbar palsy and peripheral axonal neuropathy, as early riboflavin treatment may improve or halt disease progression, thus reducing the associated mortality and morbidity.

## Introduction

Brown-Vialetto-Van Laere (BVVL) syndrome is a rare progressive motor neuron disorder with an autosomal recessive inheritance that results from a defect in riboflavin transporter genes; SLC52A2, SLC52A3 under the umbrella of recently called Riboflavin Transporter Deficiency (RTD) (1). It was first reported by Dr. Charles Henry Brown (1894) (2) as a form of familial infantile amyotrophic lateral sclerosis. Later on, further familial cases with an autosomal recessive pattern of inheritance and female predominance were reported in the literature (3, 4). Classically characterized by sensorineural hearing loss (SNHL), progressive pontobulbar palsy involving the motor components of the seventh and ninth to twelfth cranial nerves, progressive muscle weakness causing respiratory compromise, limb weakness, and upper motor signs. Disease onset usually occurs in the second decade of life, but earlier and later ages of onset have been reported (3). Symptoms, severity, and disease duration are variable. BVVL syndrome exhibits genetic heterogeneity; BVVL1 (OMIM # 211530) is caused by homozygous or compound heterozygous mutations in the SLC52A3 gene (OMIM #613350) on chromosome 20p13 (5), whereas BVVL2 (OMIM #614707) is caused by a mutation in the SLC52A2 gene (OMIM #607882) on chromosome 8q. Fazio Londe syndrome (OMIM #211500) is another RTD syndrome with a similar clinical phenotype to BVVL syndrome but without sensorineural deafness (3, 4, 6).

BVVL syndrome can mimic chronic inflammatory demyelinating neuropathy and other neuroimmune disorders, as it may present with positive cerebrospinal fluid autoantibody titers and may transiently respond to intravenous immunoglobulin (7–9). An excellent response to treatment with high doses of riboflavin was reported in the literature, with the arrest of the disease in the majority of patients and the reversal of symptom progression in some patients (5, 6, 10–12).

Herein, we report a case of BVVL1 (RTD3) with a clinical phenotype masquerading as chronic inflammatory demyelinating polyneuropathy (CIDP) at presentation, highlighting the importance of early recognition, genetic diagnosis, and riboflavin introduction to halt progression, accelerate recovery, and reduce associated morbidity and mortality.

## Case presentation

### Patient information

The patient was an 18-month-old boy who was a product of a first-degree consanguineous marriage with an uneventful pregnancy and an uncomplicated elective cesarean section at full term. He had a normal neonatal period. His developmental milestones, mainly motor and speech, were mildly delayed; he sat at age 7 months, crawled at 10 months, and stood with support at 1 year. Currently, he can stand with support and can vocalize only two words. There was no family history of spontaneous abortion or neurogenetic disorders. His father had multiple sclerosis.

He presented with insidious-onset progressive stridor (obvious during crying and feeding) and difficulty swallowing for 2 months, preceded by fever and upper respiratory tract infection (URTI) for 5 days. Afterward, he was noted to have impaired sucking, drooling of saliva, frequent choking, changes in his facial expressions, drooping of the right eyelid and less interaction with episodes of retrocollis and weight loss. He was inattentive most of the time, with a gradual decrease in verbal output and the use of nonverbal communication to indicate needs. No alteration of sensorium was observed.

At 16 months, he was evaluated by an ear, nose and throat (ENT) specialist who observed evidence of tracheal tag, subcostal retraction and biphasic stridor; however, nasopharyngeal scope, direct laryngoscopy and bronchoscopy (DLB), were unremarkable, ruling out the possibility of foreign body inhalation. Esophagoscopy was also normal.

### Clinical findings

Examination revealed an active, alert, and thriving child with normal vital signs. Intermittent soft stridor with no grunting was observed at rest. The ENT exam results were normal with no lymphadenopathy. Cranial nerve examination showed no ptosis of the left eye with normal pupils, fundus, and extraocular movements. He had bilateral lower motor neuron (LMN) facial (VII) nerve palsy, which was more pronounced on the right side; weak palatal and gag reflexes with excessive drooling; no uvular deviation; and normal tongue movements. He had a reduced response to loud sounds by simple distraction test. He had decreased strength and tone in both the upper and lower limbs and depressed deep tendon reflexes (DTRs) in the lower limbs. The plantar reflex was bilaterally flexor. His gait was unsteady with obvious hand tremors. No paradoxical abdominal movements when breathing was noted. The remainder of his systemic examination results were unremarkable (Table 1).

**Table 1:** Clinical characteristics and laboratory profile of a patient with Brown-Vialetto-Van Laere syndrome caused by a mutation in SLC52A3.

Variables	Clinical features
Sex	Male
Ethnicity	Saudi
Age at first symptoms	16 months
Consanguinity	Yes
Presenting complaint	Stridor, swallowing difficulty, and respiratory difficulty preceded by URTI. After admission, frequent choking, facial expression changes, episodes of retrocollis, inattentiveness, and progressive weakness. Needed ventilatory support for some time (Nocturnal NIV) Feeding was shifted to gastrostomy tube. Mild motor and speech delay since early infancy.
Cranial nerves	Impaired hearing, bulbar weakness, no optic atrophy
Motor	Moderate hypotonia, diffuse weakness, worse distal LL>UL; neck extension
Deep tendon reflexes	Depressed throughout; bilateral UE and LE: 1+
Cerebellar	Mild tremors, no ataxia, normal function
Gait	Did not walk previously but fell without support
Brain MRI	Bilateral smooth enhancements of the oculomotor and glossopharyngeal nerves with bilateral enhancement of the cisternal and intracanalicular segment of facial nerves.
Whole-spine MRI	Smooth enhancements of cauda equina nerve roots, predominantly involving the anterior nerve root. Neuroimaging findings likely represent polyneuropathy, possibly with a viral or autoimmune cause.
Autoimmune labs	Mildly elevated CSF protein; vasculitis work-up, negative; anti-GM-1 Ab, negative; other autoimmune Abs, negative
EMG findings	Consistent with axonal sensorimotor polyneuropathy
Riboflavin transporter gene abnormalities	Homozygous missense mutation in SLC52A3 c.211G>A (p.Glu71Lys) by WES at 18 months of age. (Gene deletion/duplication analysis negative)
Response to riboflavin titrated by response	Halted progression of symptoms with significant improvements in tone, strength, bulbar symptoms and respiratory status.
Abbreviation	NIV, non-invasive ventilation; CSF, cerebrospinal fluid; WES, whole-exome sequencing.

### Diagnostic assessment

Based on the findings of subacute-onset multiple cranial neuropathies, specifically pontobulbar palsy and impaired hearing preceded by viral illness, the diagnoses listed in (Table 2) were considered after ruling out the possibility of posterior fossa/cerebellopontine angle mass lesion.

**Table 2:** Etiology of multiple cranial nerve lesions/enhancement.

• Trauma	
• GBS (motor only)/MFS	
• CIDP	
• Mononeuritis multiplex	
• Systemic infections • Mycoplasma pneumonia • Brucellosis • EBV, CMV • Herpes virus type I	
• Chronic meningitis • Tuberculosis • Carcinoma • Sarcoidosis • Hematological malignancy (ALL)	
• Brainstem lesions • Tumors • Autoimmune related (encephalitis, ADEM, MOGAD) • Vascular	
• Paraneoplastic syndrome	
• Nasopharyngeal carcinoma	
• Arnold-Chiari malformation	

MFS, Miller Fisher syndrome; GBS, Guillain Barre syndrome; CIDP, chronic inflammatory demyelinating polyneuropathy; EBV, Epstein-Barr virus; CMV, cytomegalovirus; ALL, acute lymphoblastic leukemia; ADEM, acute disseminated encephalomyelitis; MOGAD, myelin oligodendrocyte glycoprotein antibody-associated disease.

**Figure 1:**
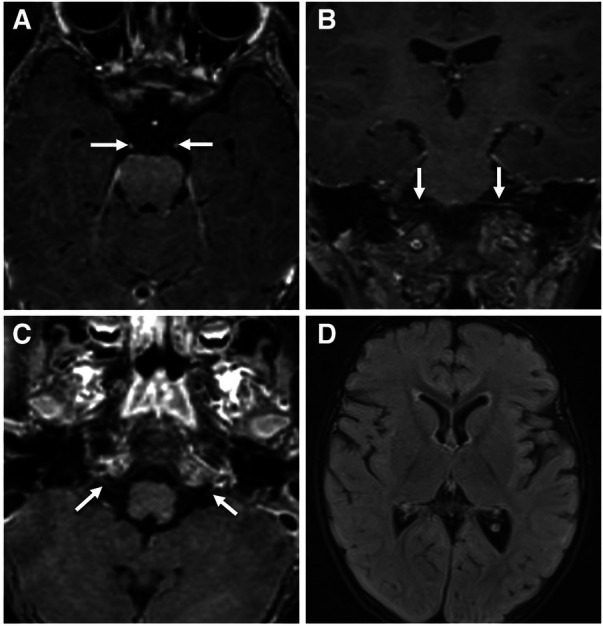
Contrast-enhanced brain MRI images acquired at 18-months of age demonstrate smooth bilateral enhancement of multiple cranial nerves, including the oculomotor nerves, cisternal and intracanalicular segments of the facial nerves and the glossopharyngeal nerves (white arrows in **A**–**C**). Axial FLAIR image (**D**) shows no signal abnormality in the brain parenchyma.

**Figure 2:**
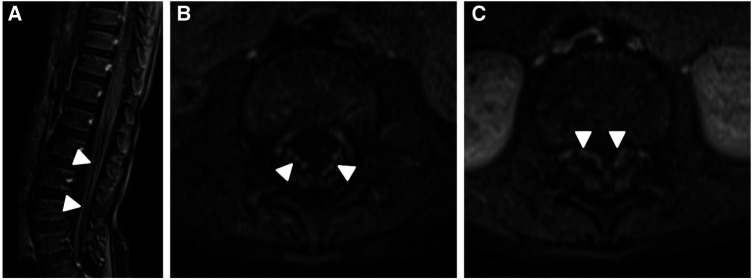
Contrast enhanced MRI of the lumbar spine obtained at the same time of the brain MRI shows smooth enhancement of the cauda equina nerve roots, particularly the anterior nerve fibres (white arrowheads in **A**–**C**).

The patient's basic hemogram, peripheral smear, and biochemistry lab results were normal. His throat swab and nasopharyngeal aspirate for common viruses were negative. Magnetic resonance imaging (MRI) with contrast revealed bilateral smooth enhancement of the oculomotor and glossopharyngeal nerves, with a possible bilateral enhancement of the cisternal and intracanalicular segments of the facial nerves (Figure 1). A whole-spine MRI with contrast showed cauda equina enhancement suggestive of chronic inflammatory demyelinating disorder (Figure 2). Cerebrospinal fluid (CSF) analysis was unremarkable with a slightly elevated protein level. The metabolic panel, including venous blood gas (VBG), serum ammonia, lactate, tandem amino acids, and urinary organic acids, was unremarkable. His thyroid panel and vitamin B12 level were normal. Anti-streptolysin O titer (ASO), mycoplasma, brucella titers, and vasculitis workup were negative. Bone marrow aspiration results were unremarkable. The findings of the electromyogram (EMG) and nerve conduction study (NCS) of the right upper and lower extremities were suggestive of a non-length dependent, predominantly axonal, sensorimotor polyneuropathy with features of ongoing denervation in the distal right upper extremity muscles (Tables 1, 3). Brain Auditory Evoked Potential (BAEP) later showed bilateral sensorineural hearing loss, while ophthalmological evaluation and visual evoked potential (VEP) were unremarkable.

**Table 3:** Summary of electromyographic (EMG) and nerve conduction study (NCS) findings in this patient at time of admission).

Nerve/sites	Latency, ms	Amplitude, mV	Segments	Distance, mm	Velocity, m/s
MNC					
R median—APB					
Wrist	2.21	1.93	Wrist-APB	70	
Elbow	4.24	1.85	Elbow-wrist	100	50
R ulnar—ADM					
Wrist	2.21	2.8	Wrist- ADM	70	
Below elbow	3.29	2.6	Below elbow-wrist	60	56
Above elbow	4.68	3.2	Above elbow-below elbow	70	50
R peroneal—EDB					
Ankle	2.00	1.66	Ankle-EDB	80	
Fibular head	4.58	1.67	Fibular head-ankle	130	50
Popliteal fossa	5.44	1.57	Popliteal fossa-fibular head	50	58
R tibial- AHB					
Medial malleolus	2.28	14.5	Medial malleolus-AHB	80	
Popliteal fossa	5.32	13.4	Popliteal fossa-medial malleolus	140	46

Nerve/sites	Onset latency, ms	Peak latency, ms	Amplitude, *μ*V	Distance, mm	Cond vel, m/s
SNC					
Median digit 2	NR	NR	NR	NR	NR
Ulnar digit 5	2.15	2.79	11.3	140	65
Sural	1.17	1.71	12.6	140	120
Superficial peroneal	NR	NR	NR	NR	NR

EMG										
Summary	Insertional activity	Spontaneous				MUAP				
Muscles		Fib	PSW	Fasc	Other	Dur	Amp	Poly	Recruit	Activation
R triceps	Normal	None	None	None		NA	NA	NA	NA	NA
R FDI	Normal	2+	2+	None		NA	NA	NA	NA	NA
R vastus lateralis	Normal	None	None	None		NA	NA	NA	NA	NA
R Tibialis anterior	Normal	None	None	None		NA	NA	NA	NA	NA
EMG										
Summary	Insertional activity	Spontaneous				MUAP				
Muscles		Fib	PSW	Fasc	Other	Dur	Amp	Poly	Recruit	Activation
R triceps	Normal	None	None	None		NA	NA	NA	NA	NA
R FDI	Normal	2+	2+	None		NA	NA	NA	NA	NA
R vastus lateralis	Normal	None	None	None		NA	NA	NA	NA	NA
R Tibialis anterior	Normal	None	None	None		NA	NA	NA	NA	NA

ADM, abductor digiti minimi; Amp, amplitude; AHB, abductor hallucis brevis; APB, abductor pollicis brevis; Cond vel, conduction velocity; EDB, extensor digitorum brevis; Fasc, fasciculation; FDI, first dorsal interosseus; Fib, fibrillation; lat, latency; MNC, motor nerve conduction; MUAP, motor unit action potential; NA, no activation; NR, no response; PSW, positive sharp wave; SNC, sensory nerve conduction. Results: Nerve conduction values are compared to pediatric NCS reference values (age group 12 to <24 months) reported by Ryan et al., (1). These reveals reduced median and ulnar compound muscle action potential amplitudes, below the 5th percentile cutoffs, with normal conduction velocities and distal latencies. Median antidromic sensory responses recording from digit 2 and superficial peroneal sensory responses are nonrecordable. Ulnar sensory responses are present but no age-adjusted reference values are available for comparison. Other motor and sensory responses in the upper and lower extremity are within normal limits. Needle EMG reveals fibrillation potentials and positive sharp waves in the first dorsal interosseous muscle, voluntary motor unit potential analysis was not possible as the patient was sedated. No fibrillation potentials could be detected in distal leg muscles. The findings are suggestive of a non-length-dependent, predominantly axonal, sensorimotor polyneuropathy with features of ongoing denervation in the distal upper extremity.

### Therapeutic intervention

The patient was started on intravenous immunoglobulin (IVIG) 2 g/kg over two consecutive days with no obvious improvement, and empirical mega-multivitamins were added, including riboflavin 200 mg/day (20 mg/kg). During his admission, he suffered arrest twice secondary to recurrent lung aspiration, received different modalities of ventilatory support and was kept in the intensive care unit (ICU) for close observation. He had a complex clinical course in the ICU for more than two months with fluctuations in his respiratory status and a need for oxygen before finalizing his diagnosis. A trial of atropine drops was administered for excessive drooling, and botulinum toxin was injected into the salivary glands. Paradoxical breathing and tachypnea were observed in the ICU. x-ray of the chest showed an eventration of the left diaphragm, and fluoroscopy confirmed diaphragmatic paralysis, probably secondary to phrenic nerve involvement, which was managed conservatively. Finally, a gastrostomy tube was inserted to minimize choking episodes. His carnitine profile showed normal levels of total carnitine, very low levels of free carnitine (7.88 μmol/L; normal range, 24.7–66.6 μmol/L) and a normal acylcarnitine profile. The possibility of motor neuron disease was considered, and the diagnosis of BVVL1 was confirmed by rapid clinical whole-exome sequencing (WES), which identified a previously reported homozygous missense mutation, NM_033409.3: c.211G>A: p. Glu71Lys, in the SLC52A3 gene (4), which resulted in the amino acid substitution of glutamic acid with lysine. No mutations were found in the SLC52A1 and SLC52A2 genes. The patient's parents were heterozygous carriers and were asymptomatic. The genetic result was published previously (13) as a part of the mass screen by Rapid WES for critically sick patients admitted to the intensive care unit. Accordingly, coenzyme Q10 supplementation was initiated, and riboflavin was increased to 400 mg (80 mg/kg/d) in two divided doses with obvious clinical improvement in facial weakness and palatal movement.

### Follow-up and outcomes

After 3 months of follow-up on riboflavin, the child had a normal gag reflex, accepted sips of water orally, and had mild facial weakness. He has started vocalizing, and subjectively, there is some response to high-frequency sounds.

**Figure 3:**
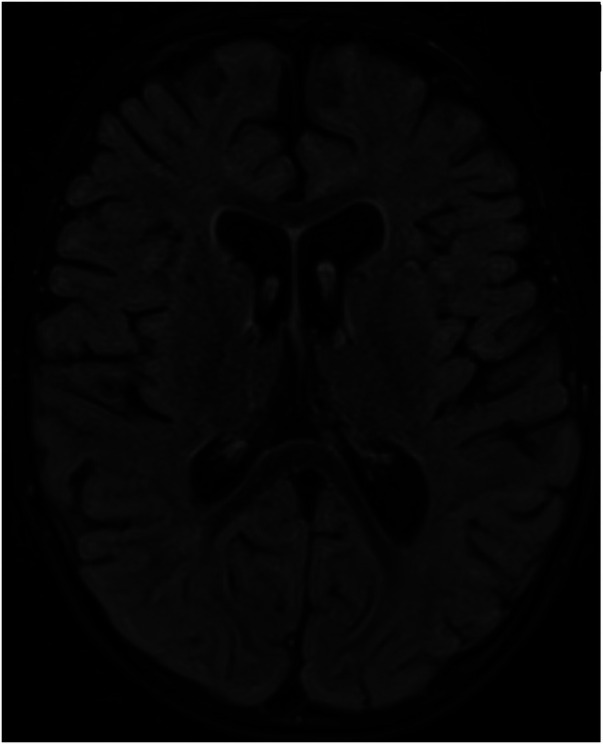
Brain MRI acquired at 19- months of age after an episode of cardiac arrest showed no evidence of hypoxic ischemic insult on the axial FLAIR image; however, there is evidence of mild brain volume loss with enlarged ventricles and prominent subarachnoid spaces.

**Figure 4:**
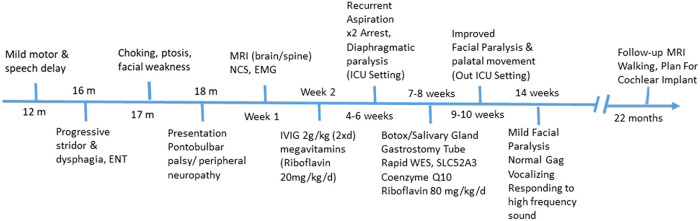
Diagnostic and patient management timeline. RTD, riboflavin transporter deficiency; CSF, cerebrospinal fluid; MRI, magnetic resonance imaging; EMG, electromyogram; NCS, nerve conduction study; WES, whole exome sequencing.

A follow-up brain MRI at 22 months showed no further enhancement of the cranial nerves or cauda equina and no evidence of acute hypoxic-ischemic insult secondary to repeated arrest, only mild cerebral atrophy (Figure 3). Clinically, he showed impressive improvement in interaction with and awareness of surroundings, facial expression, eye closure during the night, response to painful stimuli, and retention of a spontaneous cough and gag reflex. He was planned for a cochlear implant after further assessment by an ENT specialist. Figure 4 represents the timeline course for this patient.

## Discussion

Three human riboflavin transporters (RFVT) homologues have been identified: RFVT1-3 encoded by genes SLC52A1-3, respectively. SLC52A1 is mainly expressed in the placenta and intestine. SLC52A2 is ubiquitously expressed but is particularly abundant in nervous tissues. SLC52A3 is most highly expressed in testis but also intestine and prostate. Pathogenic mutations in SLC52A1 result in transient riboflavin deficiency with onset in the newborn children of mothers harboring one heterozygous SLC52A1 mutation (OMIM 615026), where the clinical symptoms are not typical of Riboflavin transporter deficiency (RTD) phenotype, and it usually subsides by 2 years of age (14). While, pathogenic mutations in the SLC52A2 and SLC52A3 (previously C20orf54) genes that encode human riboflavin transporters RFVT2 and RFVT3, respectively result in an RTD, which is a rare autosomal recessive motor neuron neurological disorder. More than one hundred RTD patients have been described to date (6).

Both Brown-Vialetto-Van Laere (BVVL) and Fazio-Londe (FL) syndromes are phenotypically continuous syndromes that lie under the umbrella of RTD caused by biallelic mutations in the human riboflavin transporter genes SLC52A2, and SLC52A3, and re-named RTD2 and RTD3, respectively (15, 16).

RTD runs a neurodegenerative course of inherited motor neuron disease with variable age of presentation, symptoms severity, and disease duration. It is characterized by both motor cranial neuropathy and sensorimotor peripheral neuropathy. Major manifestations include bulbar palsy (dysphagia, dysphonia, and tongue atrophy), facial palsy, neck and shoulder weakness, vision loss, deafness, progressive axial and distal muscle weakness, sensory ataxia, and respiratory compromise due to combined muscle weakness, and diaphragm paralysis. It is associated with high mortality in the very young age group if left untreated (10, 15, 17). Sensorineural deafness is present in BVVL only, and may not be obvious initially in twenty percent of patients (3). Other cranial nerve involvement (III, V, and VI) is less common. Less frequent manifestations include ocular abnormalities, autonomic dysfunction, epilepsy, and mental retardation (18).

Our patient had a previously reported SLC52A3 homozygous missense mutation causing clinical characteristics of RTD3, consistent with BVVL syndrome (4). Although both RTD2 and RTD3 are present early in life only RTD3 has a late onset (as late as the third decade) (19). Hearing loss, muscle weakness, and respiratory dysfunction are among the most common presenting symptoms at onset for both, but abnormal gait and/or ataxia is rarely a presenting feature of RTD3. RTD3, as in our patient commonly presents with early onset bulbar symptoms, and facial weakness whereas it is observed either later in the disease course or rarely in RTD2. Optic nerve atrophy causing nystagmus, blindness, SNHL, weakness of neck extension and upper limbs, early-onset ataxia, and sensory abnormalities are more prevalent features of RTD2 (6, 10, 18). Our patient had normal vision, but bilateral SNHL was clinically suspected based on poor attentiveness and decreased interest in the surroundings, as well as an inadequate response to various stimuli by his mother. Furthermore, he had abnormal BAEP results that showed bilateral SNHL. Based on the latest published literature, the most common clinical features of RTD3 were muscle weakness (RTD2 83%, RTD3 84%), hearing loss (RTD2 90%, RTD3 84%), respiratory distress (RTD2 53%, RTD3 73%), and bulbar palsy (RTD2 50%, RTD3 61%), usually presenting before 3 years of age (6).

In RTD neuropathy, patients predominantly present with motor neuropathy with loss of DTRs and SNHL (1), but can also present with predominantly sensory symptoms (20). The exact pathophysiology of RTD-related neuropathy is not known, but a mitochondrial dysfunction has been postulated as riboflavin is a precursor for several metabolites that are involved in the electron transport chain (5). A preceding viral illness or an infection with fever has been reported to precipitate the disease onset in some genetically predisposed patients (7, 21). Based on this observation, infectious or autoimmune factors have been postulated to play a role in the presentation of RTD-related neuropathy. Motor and sensory nerve conduction studies (NCS), and electromyogram (EMG) in most RTD cases will reveal an axonal rather than demyelinating neuropathic phenotype, with signs of anterior horn dysfunction, and chronic denervation. RTD3 cases may have normal NCS, and EMG results initially (6). Our patient had similar findings of sensorimotor abnormalities and predominantly axonal polyneuropathy with features of ongoing denervation.

MRI is usually normal in RTD (16), but RTD3 patients specifically may have hyperintense T2-weighted signals within cerebellar peduncles (22), cortical, subcortical (basal ganglia and internal capsule), and brainstem (vestibular nuclei and central tegmental tract) regions (22–24). In contrast, RTD2 may have mild atrophy of the cerebellar vermis (25), optic nerve abnormalities (26), and thinning/shortening of the corpus callosum (26). In general, Spinal MRI in RTD may show abnormal T2-weighted intensities in ventral nerve roots and dorsal regions of the spinal cord (22, 23, 27, 28). MRI in our patient showed multiple cranial nerves (3rd, 7th, and 9th) and cauda equina enhancement, not consistent with the expected changes seen on the brain MRI of RTD3 patients.

Acute inflammatory demyelinating polyradiculoneuropathies (AIDPs), autoimmune axonal motor neuropathy, juvenile-onset amyotrophic lateral sclerosis, myasthenia gravis, and mitochondrial myopathy should be considered in the differential diagnosis of RTD, especially in the presence of bulbar weakness, and absence of SNHL. Several patients received intravenous immunoglobulin (IVIG) and steroids prior to receiving autoantibody testing results (7, 29, 30), with some responding at least transiently to immunotherapies (7, 21). A unique response to riboflavin supplementation with reversal of some symptoms is a key difference distinguishing RTD from other disorders.

Based on the constellation of our patient's symptoms, and signs, a prodrome of viral illness, duration of illness, initial MRI findings suggesting acquired inflammatory autoimmune polyneuropathy and elevated CSF protein, Chronic inflammatory demyelinating polyneuropathy (CIDP) was suspected, and he was treated with IVIG accordingly. No improvement was observed, and as his autoimmune, vascular, and paraneoplastic panel results were negative, a trial of megavitamins including riboflavin was initiated at 20 mg/kg/day, but he responded to higher doses of 50 mg/kg/day, which was titrated further to 80 mg/kg/day after genetic confirmation of BVVL1, and initial stabilization of his rapidly progressive weakness, bulbar palsy, and the respiratory compromise was achieved on this dose.

Our patient is unique in his presentation compared with the three patients reported previously by Allison T, et al. (8), who had progressive weakness secondary to CIDP. The main manifestation in our patient was pontobulbar palsy with respiratory compromise rather than hypotonia or progressive peripheral weakness. One of the previously reported patients, a 3-year-old child, had a positive anti-GM1 antibody test (1:100), consistent with autoimmune-mediated motor neuropathy, which may have made it more difficult to associate hearing loss with the disease entity causing muscle weakness (8).These findings had been reported previously in this syndrome and led to an initial diagnosis of multifocal motor neuropathy (9) with a transient response to steroid and/or intravenous immunoglobulin treatment, which falsely confirmed the suspicions of autoimmune disorders (7, 9).

Multiple factors can potentially result in missing a treatable RTD, including positive serology for autoimmune antibodies (anti-LGl1, anti-GM1), clinical response to intravenous immunoglobulin, and lack of any history of hearing loss at presentation, which only occurs in approximately two-thirds of patients with RTD with onset prior to age 4.

Our patient had an initial low free carnitine level, while the plasma acylcarnitine profile was normal. Urine organic acid analysis was suggestive of ethylmalonic acidemia. These were consistent with the previously reported metabolic abnormalities in BVVL that occur secondary to impaired riboflavin absorption, which includes multiple acyl-CoA dehydrogenation defects (MADD) like- picture with elevated plasma acylcarnitine secondary to an impairment in the metabolism of fatty acids by mitochondrial β-oxidation, and urine organic acid analysis abnormalities in nearly half of the RTD cases. Ethylmalonic aciduria suggestive of impairments in fatty acid, methionine, and/or isoleucine oxidation is the most common abnormality noted (RTD2 4/10, RTD3 5/12) (6).

RTD is a treatable genetic disorder, where maintaining a high index of suspicion, and early institution of riboflavin is crucial to stop disease progression and reverse some clinical manifestations until another diagnosis has been confirmed or genetic testing for RTDs has returned negative results. Early high-dose oral riboflavin supplementation is effective in preventing mortality, and the need for longer ventilatory support as well as reducing morbidity related to deficits in cranial nerves, muscle strength, motor abilities, and respiratory function in over 70% of patients (10). Effective doses vary between 10 and 80 mg/kg /kg body weight per day, and titration of the dose is recommended based on the best patient`s response (6, 11, 12). A small dose of riboflavin 20 mg/kg/day was not effective with our patient as reported previously (1), but a high dose of 80 mg/kg/day was effective in reversing multiple deficits and maintaining a steady improvement over months. He started to walk while supported, and his speech as well as his ability to talk and swallow also improved. There was a subjective improvement only in hearing, and eventually, he needed cochlear implantation. The response and persistence of hearing loss posttreatment have not been well-studied in patients with BVVL syndrome, and the results of cochlear implants have not been encouraging (31). Interestingly, post-therapy the follow-up MRI showed the disappearance of previous findings, and the presence of mild generalized atrophy probably not related to hypoxia but similar to previously reported findings of the brainstem, and cerebellum atrophy detected in some patients with BVVL syndrome (32).

The disease has a variable course and prognosis, which is also influenced by the timing of initiation of riboflavin treatment. High-dose riboflavin was reported to produce clinical improvement in BVVL syndrome (30), and prolongation of survival (1). Without riboflavin treatment, a more severe disease course, irreversible morbidity, mainly quadriparesis and hearing loss, and early death were reported in those with younger age at presentation; the mean time to death was reported to be approximately 10 months in those presenting before 3 years of age, while the mean time to death was approximately 13 years in children presenting from 3 to 18 years of age even in patients with similar genetic mutations, probably due to unknown altered pathophysiological mechanisms.

In conclusion, although BVVL syndrome (riboflavin transporter disorders) is a rare inherited disorder, it should be suspected in the presence of pontobulbar palsy, progressive weakness, respiratory compromise, and motor cranial neuropathy even in the absence of hearing loss. Although riboflavin trials can help in early clinical diagnosis, mutational molecular analysis can confirm the genetic diagnosis and facilitate early lifesaving riboflavin treatment, genetic counseling, and future family planning.

## Data Availability

The original contributions presented in the study are included in the article/Supplementary Material, further inquiries can be directed to Amal Y. Kentab, akentab@ksu.edu.sa.
